# Megahertz scan rates enabled by optical sampling by repetition-rate tuning

**DOI:** 10.1038/s41598-021-02502-w

**Published:** 2021-11-26

**Authors:** D. Bajek, M. A. Cataluna

**Affiliations:** School of Engineering and Physical Sciences, Heriot-Watt University, Edinburgh, EH14 4AS UK

**Keywords:** Optics and photonics, Lasers, LEDs and light sources, Ultrafast photonics, Electronics, photonics and device physics

## Abstract

We demonstrate, for the first time, optical sampling by repetition-rate tuning (OSBERT) at record megahertz scan rates. A low-cost, tunable and extremely compact 2-section passively mode-locked laser diode (MLLD) is used as the pulsed laser source, whose repetition rate can be modulated electronically through biasing of the saturable absorber section. The pulsed output is split into two arms comparable to an imbalanced Michelson interferometer, where one arm is significantly longer than the other (a passive delay line, or PDL). The resulting electronic detuning of the repetition rate gives rise to a temporal delay between pulse pairs at a detector; the basis for time-resolved spectroscopy. Through impedance-matching, we developed a new system whereby a sinusoidal electrical bias could be applied to the absorber section of the MLLD via a signal generator, whose frequency could be instantly increased from sub-hertz through to megahertz modulation frequencies, corresponding to a ground-breaking megahertz optical sampling scan rate, which was experimentally demonstrated by the real-time acquisition of a cross-correlation trace of two ultrashort optical pulses within just 1 microsecond of real time. This represents scan rates which are three orders of magnitude greater than the recorded demonstrations of OSBERT to date, and paves the way for highly competitive scan rates across the field of time-resolved spectroscopy and applications therein which range from pump probe spectroscopy to metrology.

## Introduction

### Optical sampling techniques

Applications in time-resolved spectroscopy range from life-sciences^1^ to metrology^2^. Optical sampling techniques and their development are therefore driven by a variety of technical demands. The choice of technique (and pulsed laser source) will influence not only the cost and footprint of the system, but the scanning parameters such as temporal resolution (step-size), maximum scan range and of course, scan rate. In particular, pump-probe spectroscopy is the process of exciting a sample of interest with one energetic ultrashort pulse of light, before investigating its response using a second, temporally delayed, probe pulse. The probe pulse can then be incrementally delayed with respect to the pump pulse in order to examine the sample’s behaviour over a temporal scan range. Smaller step-sizes will lead to higher resolution scans, longer scan ranges will allow for longer events to be scanned, and fast scan rates will allow for faster acquisitions. Among the state-of-the-art is ASOPS^3–6^—asynchronous optical sampling—which uses two lasers with a slight difference in repetition rate. When directed towards a target of interest, this offset leads to an increasing delay between pulse pairs, in such a manner that one scan may be completed with a scan range equal to the roundtrip period of one of the lasers, and at a scan rate equivalent to the offset in repetition rates. Often demonstrated using expensive solid-state lasers, despite requiring no moving parts, ASOPS comes with the clear drawbacks of requiring two such lasers, and technical intricacy surrounding the electronic timing and locking of the repetition rates. To date, ASOPS has been demonstrated to kHz scan rates^7^. On the other hand, OSCAT^8^—Optical Sampling By Cavity Tuning—uses only one laser in a highly imbalanced Michelson interferometer setup, where the scan is driven not by mechanical variations in the lengths of one of the arms, but by mechanical variations in the laser cavity length itself. It was therefore demonstrated that optical sampling can occur in such an arrangement by modulating the laser’s repetition rate. Only one high-end laser is typically used for this technique, but it is of course limited by physically moving parts. To date, scan rates of a few hundred Hz have been demonstrated^9,10^. PHIRE (parallel heterodyne interferometry via rep-rate exchange), a conceptually similar single-laser technique, was demonstrated for use in dual-comb interferometry^11^, where kHz scan rates were achieved (164 kHz in the case of raw acquisitions, slowed to a few kHz when averaging was used to improve the signal quality). PHIRE has been designed to function similarly to a single-laser alternative to the two-laser ASOPS technique, and does so by rapid switching of the repetition rate from one value to another, whereas OSBERT makes use of OSCAT-type scanning, which means actively tuning the repetition rate through multiple discrete values of repetition rate, each one giving rise to a scan point in the acquisition. In addition, PHIRE is fairly technically complex, requiring various external and electronic components to function, including two phase-coherent RF synthesizers, Electro-Optical Modulators and Acousto-Optic Modulators. Furthermore, this method required a substantial imbalance in the interferometer arms, using a 600 m fibre optic cable as a passive delay line similar to those used in OSCAT. Longer passive delay lines lead to a number of adverse effects, including both dispersion of the pulses and cumulative pulse-to-pulse jitter, which will greatly affect the cross-correlated signal, especially for high resolution scans or sub-picosecond pulses which are often employed for non-linear interactions.

The crucial difference between OSBERT and these techniques is that we take advantage of entirely electronically driven scanning, which is enabled by applying an electronic bias directly to the metal contacts of our unique choice of laser: the 2-section quantum dot semiconductor mode-locked laser diode (MLLD). Since these are diodes, they are entirely electronically pumped, meaning there is no requirement for optical pumping by a second laser which is commonplace for the solid state and fibre-laser alternatives. Compared to such benchtop lasers which can take up considerable surface area on an optical bench, these compact devices are on the order of millimetres long, and can be directly implemented into circuitry components, being of similar size. MLLDs are also extremely low cost, where multiple devices can be made from the growth of a single semiconductor wafer at a fraction of the price of commercial alternatives. The output characteristics of MLLDs can also therefore be tuned simply by varying the applied biasing levels, including their power, repetition rate, pulse duration and wavelength to various degrees, without the need for external components or additional modules. Finally, in contrast to the cutting edge described above, OSBERT requires no mechanical parts, no external optical components, and no complex electronics when an MLLD is used as the laser source in optical sampling. It is these key advantages that allow us to acquire traces at megahertz scan rates, which to the best of our knowledge is the first time in which optical sampling of this nature has been conducted at such speeds.

### The mode-locked laser diode and OSBERT

**Figure 1:**
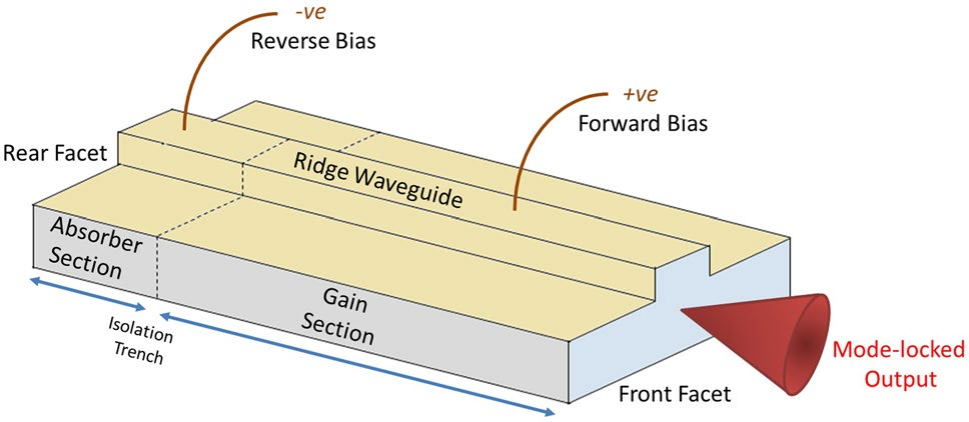
Typical 2-section MLLD, featuring a ridge waveguide, and two electronically isolated sections; the saturable absorber section and the gain section.

2-Section mode-locked laser diodes are compact semiconductor lasers which are utilized for their ability to provide a variety of output characteristics^12–14^ which can be modulated by simple control of their electrical biasing conditions. Passive mode-locking, which leads to the generation of ultrashort pulses, may be achieved by forward biasing a gain section which is electrically isolated from a reverse-biased saturable absorber section, see Fig. 1.

It is well understood from the literature and our previous reports^15,16^ that small variations in the driving conditions (temperature, forward bias and reverse bias) allow for the tuning of the laser output, notably the repetition rate^17–19^. Varying the biasing levels of the far shorter absorber section in order to tune the repetition rate is a principle OSBERT takes advantage of in order to deliver optical sampling, which is similar to that of OSCAT, but without the need for any moving parts whatsoever. Various discussions are available in the literature as to the underlying principles behind this tuning, where several mechanisms may all contribute to the tuning at the same time. These include the Pockels’ effect, which may lead to variations in the semiconductor material refractive index and therefore roundtrip period^18^. Similarly, the plasma effect has been cited as a contributor^20,21^, although the change here in refractive index arises due to a change in carrier density^22^. The Quantum Confined Stark Effect^23,24^ also describes the effect of an external electric field on the light emission or absorption spectrum of quantum wells, wires or dots. We consider that a number of these and other complex carrier dynamic effects contribute to a variety of tuning (in both directions and magnitude) in devices of differing composition and structures, and in this case, we take advantage of this novelty to demonstrate optical sampling without moving parts.

The maximum scan range $\Delta \tau$ of the OSBERT technique is scaled by the total length of imbalance in the passive delay line (PDL) and by the range of repetition rate tunability $\Delta f$, such that

1 $$\Delta \tau = \frac{{l \cdot n \cdot f_{min} }}{c}\left( {\frac{1}{{f_{min} }} - \frac{1}{{f_{min} + \Delta f}}} \right)$$

where $f_{min}$ is the initial repetition rate of the laser, *n* is the refractive index of the medium of the PDL, and *c* is the speed of light in a vacuum. As such, a δ-step increment in $\Delta f$, which is achieved by a δ-step increment in the voltage supplied to the absorber section, in this configure gives a δ-step increment within the scan range. Our previous work^16^ showcased the first acquisition of optical pulse cross-correlation traces at 1 kHz scan rates using the OSBERT technique. This was then utilised in a metrology application as a distance measurement, where target’s motion across 13.0 mm was detected with ~ 0.1 mm standard deviation from an equivalent free-space distance of 36 m.

To the best of our knowledge, the following work demonstrates optical sampling by electronic repetition-rate tuning at ground-breaking megahertz scan rates, enabled by low-cost, compact and versatile 2-section mode-locked laser diodes.

## Methods

### Experimental setup

**Figure 2:**
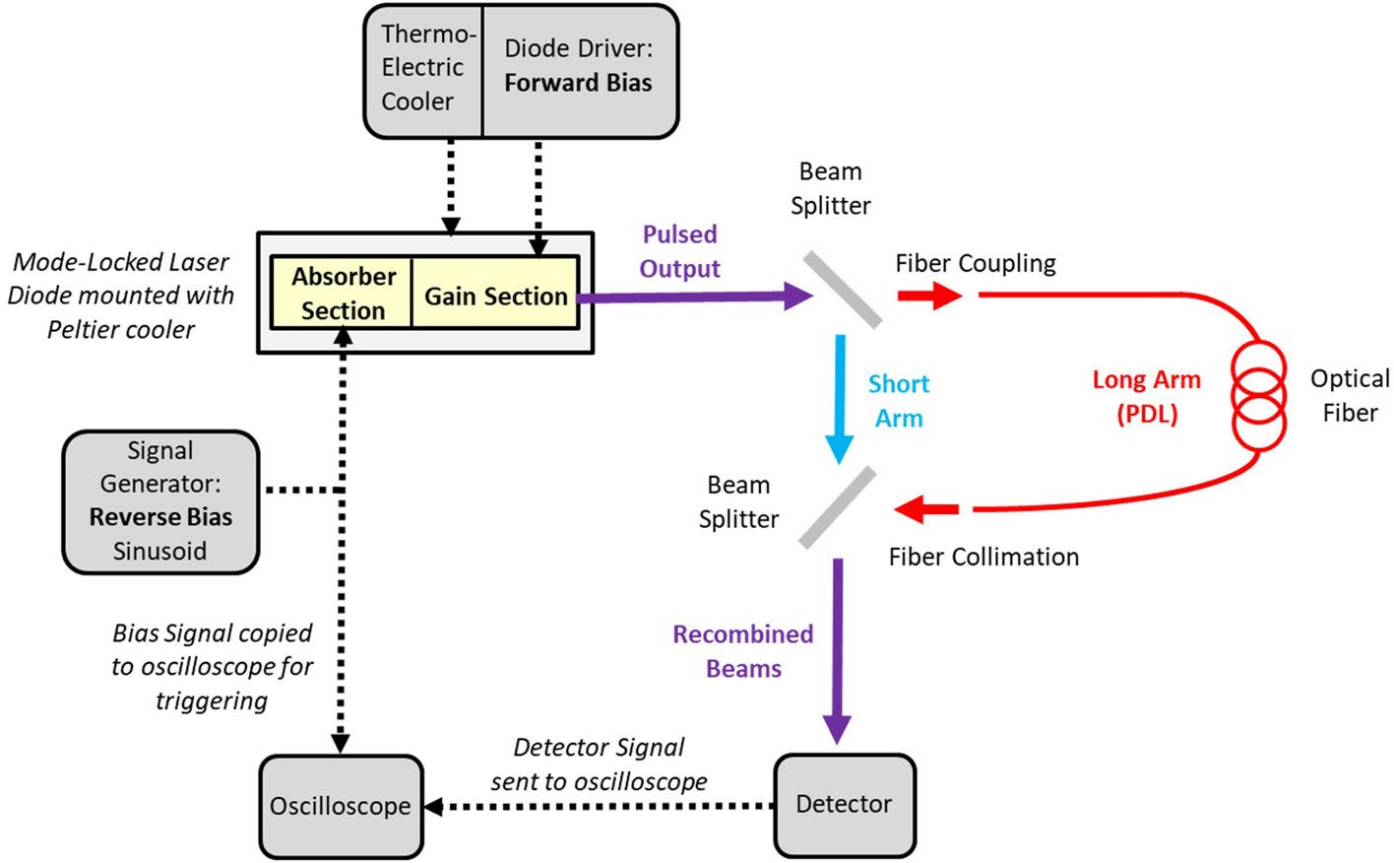
The OSBERT setup used to acquire cross-correlation traces at megahertz scan rates.

The experimental setup is shown in Fig. 2, and is based on that found in our previous work^16^, including the same MLLD whose fabrication and characteristics may be found there. The forward current supplied to the gain section of the MLLD was held constant throughout all experiments at 210 mA, whilst the entire device temperature was held constant at 20 °C. By reverse biasing the saturable absorber section, few-picosecond pulses of wavelength 1260 nm were generated at an initial repetition rate of 5.079 GHz, and as previously described, was found to be electronically tunable by approximately 10 MHz by varying the reverse bias applied. This pulsed output was then split in to the two arms of the imbalanced interferometer: a free-space short arm and a long arm consisting of a single-mode optical fibre passive delay line (PDL) of length *l* = 5 m and refractive index *n* = 1.46.

The arms are arranged in such a way that the free-space length of the short arm is equal to the sum of free-space required to both couple and then collimated the beam before and after the optical fibre respectively—therefore on balance, the PDL length is determined by the length of the optical fibre alone. Both pulse trains then meet at the second beam-splitter wherein their pulses are temporally delayed with respect to each other.

### Acquisition of cross-correlation traces

The recombined beams are directed to the fast photodetector (*Thorlabs InGaAs DET08CL/M, 5 GHz bandwidth)*, whose output signal is then received at an oscilloscope (*Teledyne Lecroy HDO4104, 1 GHz bandwidth)*. The saturable absorber bias modulation which gives rise to repetition rate tunability is achieved using a signal generator (*Keysight Technologies 33612A Waveform Generator, 80 MHz*), a copy of which is used to trigger the oscilloscope. A sinusoidal signal drives the continuous repetition rate tuning of the MLLD by setting a voltage offset (*V*_*off*_ =  − 6.80 V) and voltage amplitude (*V*_*pp*_ = 1.20 V), which were previously found to give rise to ~ 3.4 MHz repetition rate tunability^16^. Using expression 1, this gives a scan range $\Delta \tau$ of ~ 16.3 picoseconds, which was more than sufficient to fully capture the duration of a cross-correlation trace of the two pulse copies interfering at the detector.

## Results

**Figure 3:**
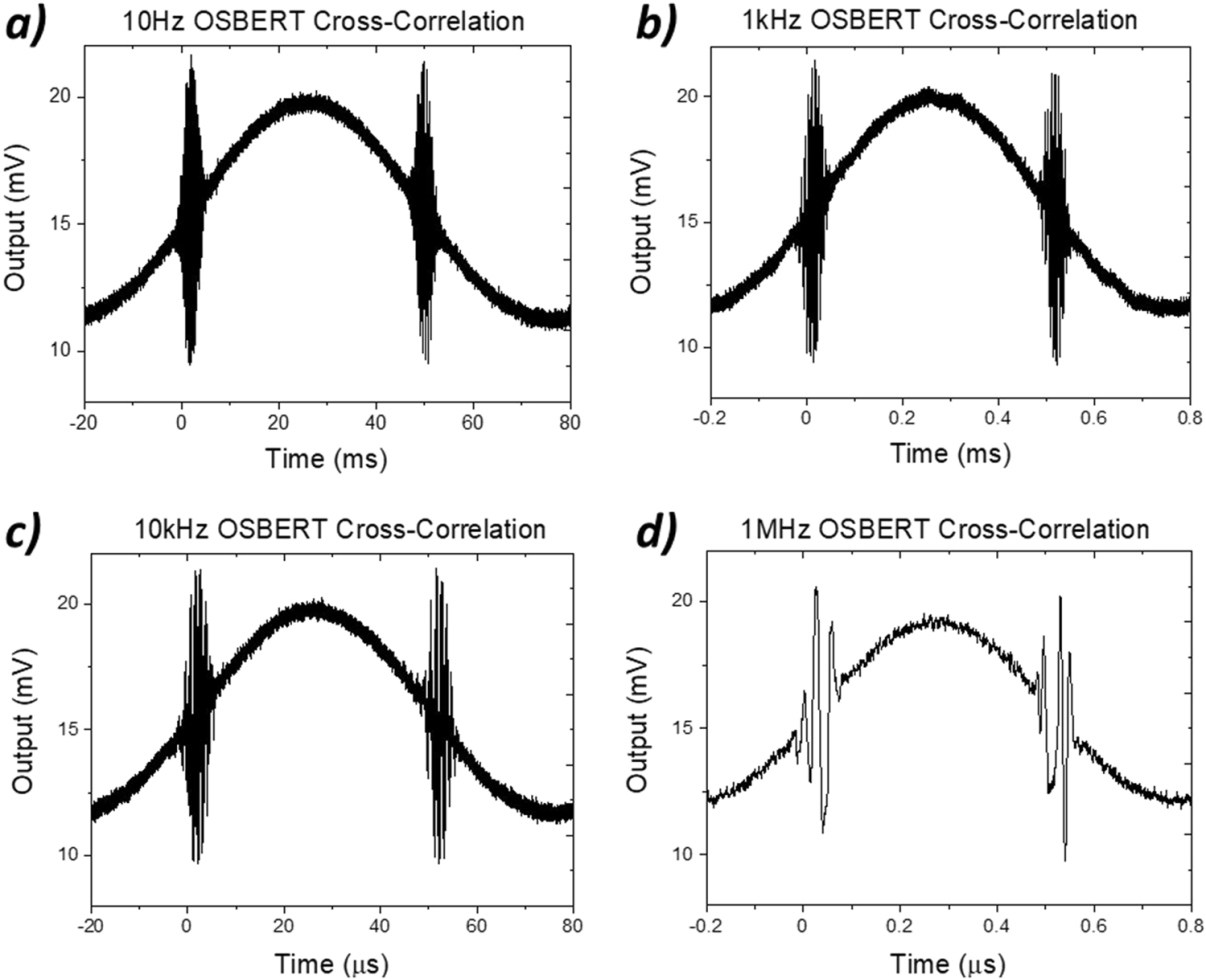
Live oscilloscope traces showing 10 Hz, 1 kHz, 10 kHz and 1 MHz OSBERT cross-correlation traces featured over a real time duration of 100 ms, 1 ms, 100 µs and 1 µs respectively.

We acquire live and unaltered scans at rates over four orders of magnitude; firstly a 10 Hz OSBERT scan, followed by 1 kHz, 10 kHz and 1 MHz. By increasing the frequency at the signal generator, we instantaneously increase the scan rate of the system. In each case, a full sinusoidal period of scanning is acquired on the time-base of the oscilloscope, which includes both a forward and a reverse scan (trough to peak followed by peak to trough), see Fig. 3.

For the 10 Hz scan, the time-base of the live oscilloscope trace shows the scan completing over the course of 100 ms. The experiment was then repeated under the exact same parameters, but with the modulation frequency increased to 1 kHz, which is reflected in the time-base which clearly shows a live acquisition taking place over 1 ms. We noted little discernible difference between 10 Hz and 1 kHz cross correlation scans, and that we were able to do so instantaneously by simply increasing the driving frequency at the signal generator without any adjustment or alignment of the system. The scan rate is then again seamlessly increased to show scans acquired at 10 kHz corresponding to 100 μs, where a reduction of the number of fringes present in the cross-correlation was noted. Finally, the scan rate was increased at the signal generator to show scans acquired at the maximum 1 MHz, where the number of discernible fringes reduced. Thus, for the first time, optical sampling was conducted over just 1 μs using the OSBERT technique, where the cross-correlation of picosecond pulses was acquired at a scan rate of 1 MHz.

## Discussion

Across all traces at all scan rates from 10 Hz to 10 MHz, what is clear is the overall shape of the traces (amplitude and width) remains centred within the scan. What changes is simply the quality of the trace in terms of the number of distinguishable fringes of each. This significant reduction in the number of fringes within the cross-correlation may be explained partially due to the 1 GHz bandwidth limitations of the oscilloscope contributing to a Nyquist effect in resolving the rise-times associated with the cross-correlation fringes at faster scan rates, which could be overcome with a system of greater bandwidth. Additionally, we must consider the number of pulses present during a cross-correlation scan. Given the repetition rate of the narrow-ridge device is approximately 5 GHz, this means approximately 5 billion pulse pairs are cross-correlated during each real-time second of acquisition. Extending this concept, only 500 million pulse pairs are present during the 10 Hz scan, whilst a mere 5000 pulse pairs are present during the entirety of the 1 MHz cross-correlation, which impedes the ability to resolve quite as many fringes. Therefore, we consider that overall, faster detection systems would allow us to resolve more features, though slower scan rates will allow us to retain a greater resolution in the time-step. Traces acquired at faster scan rates (such as those seen at 1 MHz) are still applicable to simple metrology measurements, where the detection of any traces is sufficient to detect the location of a target. This is particularly true if we were to reconfigure our setup to acquire a non-linear signal, such as a simple intensity second-harmonic signal from a non-linear crystal such as that shown in the OSCAT technique^8^. Further investigation would allow us to significantly optimise the system for various sensing scenarios after implementation of these improvements.

**Figure 4:**
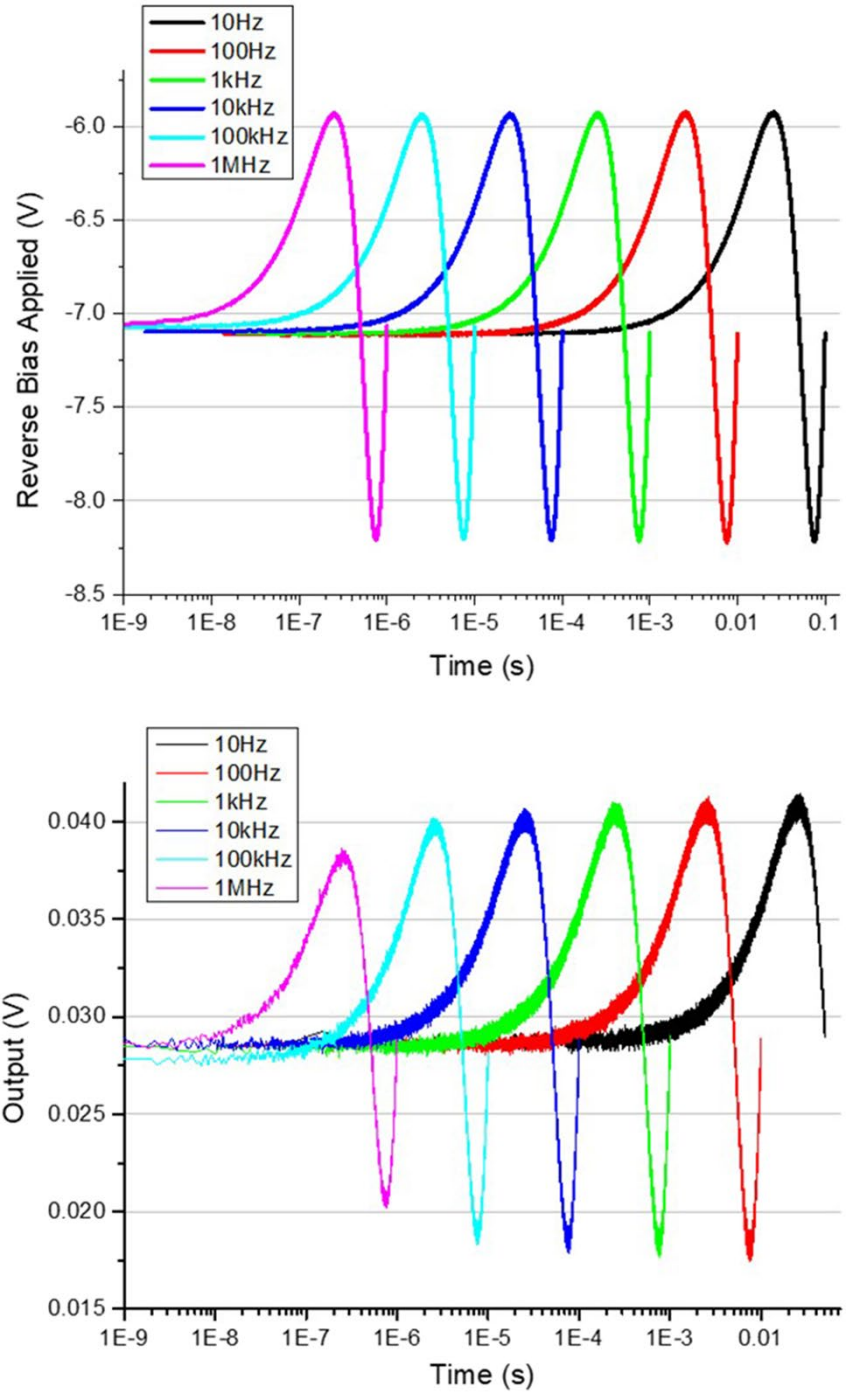
Above, the reverse bias signal applied to the absorber section of the MLLD across several modulation frequencies. Below, the detector output across the same modulation frequencies.

Finally, we consider the complex physical dynamics which are at play whilst electronically modulating one section of the MLLD, for which we currently limited in our understanding. In order to gather some information regarding this, an OSBERT scan was set up which disallowed the opportunity for a cross correlation trace centred in the scan. The output of the detector is then sent to the oscilloscope, along with a copy of the original input modulation signal from the signal generator, see Fig. 4, for various scan rates.

What is immediately evident is that whilst the input biasing signal maintains its form and amplitude up to modulation speeds of 1 MHz, the same cannot be said for the apparent output performance of the MLLD. After approximately 1 kHz modulation speeds, the device begins to show signs of degraded response to the input signal, as the amplitude from the output begins to drop. As such, if there is a reduction in the extremes of the output sinusoid, this suggests firstly that the extremes in maximum and minimum average power have not been electrically realised, which could signify that the extremes in maximum and minimum reverse bias have not been realised either. Since the biasing levels are also related to the repetition rate tunability, this could mean the tunability has somehow reduced due to faster modulations.

The cause of the reduction in the amplitude of the repetition rate tunability for faster scan rates is not understood at present. It is possible that the fast modulation of the small absorber section compared to the statically biased larger gain section leads to discrepancies in the temperature of the active region, which is also known to influence the repetition rate. Such a complex interplay of these dynamics would be a priority for future study. It is also possible that higher quality driving equipment is required, such as shortening and replacing the SMA cables between the signal generator and the absorber section with higher bandwidth alternatives. Since these traces are presented in their raw, real-time form, we consider that some standard signal processing techniques, such as time-averaging the traces, could also improve their quality but at the expense of increased acquisition time (and therefore lower scan rates).

In conclusion, the potential of the system is well highlighted in this work. For the first time, optical cross-correlations from a two-section quantum dot mode-locked laser diode were detected and acquired at up to megahertz scan rates, using only a single laser, with absolutely no moving parts, demonstrating the proof of concept of continuous scanning by applying an electrical signal to the absorber section of the diode in the context of an imbalanced interferometer. These novel semiconductor devices represent an extremely low-cost, low-footprint and versatile substitute for the conventional choice of bulky, complex and expensive commercial alternatives such as solid-state systems. Moreover, compared to two-laser techniques such as ASOPS, OSBERT may be conducted using only one such device. An optimised OSBERT system would therefore be a significant competitor for time-resolved spectroscopy applications across life-sciences and metrology.

## Data Availability

The datasets generated during and/or analysed during the current study are available from the corresponding author on request.
